# Supplementary material for: Pyruvate Dehydrogenase Kinase 1 inhibition mediated oxidative phosphorylation enhancement in cartilage promotes osteoarthritis progression
Source: BMC Musculoskelet Disord. 2023 Jul 20;24:597. doi: 10.1186/s12891-023-06585-6 (PMC10357736; doi:10.1186/s12891-023-06585-6)
Supplement: Supplementary file 1 — Additional file 1. [file 12891_2023_6585_MOESM1_ESM.zip › BP.Gsea.1653623667859/GOBP_ENDODERM_DEVELOPMENT.html]

Details for gene set GOBP\_ENDODERM\_DEVELOPMENT[GSEA]

|  || Dataset | 55457\_collapsed\_to\_symbols.cls.cls #OA\_versus\_normal.cls.cls #OA\_versus\_normal\_repos |
| Phenotype | cls.cls#OA\_versus\_normal\_repos |
| Upregulated in class | normal |
| GeneSet | GOBP\_ENDODERM\_DEVELOPMENT |
| Enrichment Score (ES) | -0.44085866 |
| Normalized Enrichment Score (NES) | -1.7856029 |
| Nominal p-value | 0.0032 |
| FDR q-value | 0.10847957 |
| FWER p-Value | 0.987 |
Table: GSEA Results Summary

  

Fig 1: Enrichment plot: GOBP\_ENDODERM\_DEVELOPMENT      
 Profile of the Running ES Score & Positions of GeneSet Members on the Rank Ordered List

  

| SYMBOL | TITLE | RANK IN GENE LIST | RANK METRIC SCORE | RUNNING ES | CORE ENRICHMENT || 1 | SMAD2 | SMAD family member 2 [Source:HGNC Symbol;Acc:HGNC:6768] | 275 | 0.768 | 0.0089 | No |
| 2 | VTN | vitronectin [Source:HGNC Symbol;Acc:HGNC:12724] | 297 | 0.753 | 0.0369 | No |
| 3 | COL8A1 | collagen type VIII alpha 1 chain [Source:HGNC Symbol;Acc:HGNC:2215] | 411 | 0.702 | 0.0558 | No |
| 4 | ITGB2 | integrin subunit beta 2 [Source:HGNC Symbol;Acc:HGNC:6155] | 827 | 0.562 | 0.0456 | No |
| 5 | HSBP1 | heat shock factor binding protein 1 [Source:HGNC Symbol;Acc:HGNC:5203] | 830 | 0.560 | 0.0675 | No |
| 6 | ITGA4 | integrin subunit alpha 4 [Source:HGNC Symbol;Acc:HGNC:6140] | 1854 | 0.379 | 0.0028 | No |
| 7 | COL11A1 | collagen type XI alpha 1 chain [Source:HGNC Symbol;Acc:HGNC:2186] | 2128 | 0.347 | -0.0048 | No |
| 8 | MED12 | mediator complex subunit 12 [Source:HGNC Symbol;Acc:HGNC:11957] | 2588 | 0.292 | -0.0291 | No |
| 9 | BMPR1A | bone morphogenetic protein receptor type 1A [Source:HGNC Symbol;Acc:HGNC:1076] | 2745 | 0.275 | -0.0304 | No |
| 10 | ITGB5 | integrin subunit beta 5 [Source:HGNC Symbol;Acc:HGNC:6160] | 2823 | 0.267 | -0.0259 | No |
| 11 | GATA4 | GATA binding protein 4 [Source:HGNC Symbol;Acc:HGNC:4173] | 3009 | 0.249 | -0.0305 | No |
| 12 | EXT1 | exostosin glycosyltransferase 1 [Source:HGNC Symbol;Acc:HGNC:3512] | 3321 | 0.219 | -0.0461 | No |
| 13 | ONECUT1 | one cut homeobox 1 [Source:HGNC Symbol;Acc:HGNC:8138] | 3443 | 0.207 | -0.0473 | No |
| 14 | LAMB3 | laminin subunit beta 3 [Source:HGNC Symbol;Acc:HGNC:6490] | 3679 | 0.188 | -0.0582 | No |
| 15 | SMAD3 | SMAD family member 3 [Source:HGNC Symbol;Acc:HGNC:6769] | 4061 | 0.150 | -0.0820 | No |
| 16 | KIF16B | kinesin family member 16B [Source:HGNC Symbol;Acc:HGNC:15869] | 4355 | 0.124 | -0.1000 | No |
| 17 | FGF8 | fibroblast growth factor 8 [Source:HGNC Symbol;Acc:HGNC:3686] | 4761 | 0.090 | -0.1280 | No |
| 18 | COL5A2 | collagen type V alpha 2 chain [Source:HGNC Symbol;Acc:HGNC:2210] | 5262 | 0.051 | -0.1649 | No |
| 19 | COL5A1 | collagen type V alpha 1 chain [Source:HGNC Symbol;Acc:HGNC:2209] | 5414 | 0.039 | -0.1752 | No |
| 20 | GATA6 | GATA binding protein 6 [Source:HGNC Symbol;Acc:HGNC:4174] | 5537 | 0.030 | -0.1835 | No |
| 21 | CDC73 | cell division cycle 73 [Source:HGNC Symbol;Acc:HGNC:16783] | 5604 | 0.023 | -0.1877 | No |
| 22 | MMP8 | matrix metallopeptidase 8 [Source:HGNC Symbol;Acc:HGNC:7175] | 5631 | 0.021 | -0.1889 | No |
| 23 | PAF1 | "PAF1 homolog, Paf1/RNA polymerase II complex component [Source:HGNC Symbol;Acc:HGNC:25459]" | 5901 | 0.002 | -0.2098 | No |
| 24 | MACROH2A1 | macroH2A.1 histone [Source:HGNC Symbol;Acc:HGNC:4740] | 5953 | -0.002 | -0.2137 | No |
| 25 | LHX1 | LIM homeobox 1 [Source:HGNC Symbol;Acc:HGNC:6593] | 6164 | -0.017 | -0.2294 | No |
| 26 | DKK1 | dickkopf WNT signaling pathway inhibitor 1 [Source:HGNC Symbol;Acc:HGNC:2891] | 6392 | -0.034 | -0.2457 | No |
| 27 | CTR9 | "CTR9 homolog, Paf1/RNA polymerase II complex component [Source:HGNC Symbol;Acc:HGNC:16850]" | 6430 | -0.037 | -0.2471 | No |
| 28 | GDF3 | growth differentiation factor 3 [Source:HGNC Symbol;Acc:HGNC:4218] | 6757 | -0.060 | -0.2702 | No |
| 29 | HMGA2 | high mobility group AT-hook 2 [Source:HGNC Symbol;Acc:HGNC:5009] | 6850 | -0.067 | -0.2747 | No |
| 30 | SETD2 | "SET domain containing 2, histone lysine methyltransferase [Source:HGNC Symbol;Acc:HGNC:18420]" | 6926 | -0.074 | -0.2776 | No |
| 31 | MMP2 | matrix metallopeptidase 2 [Source:HGNC Symbol;Acc:HGNC:7166] | 7037 | -0.082 | -0.2829 | No |
| 32 | ITGAV | integrin subunit alpha V [Source:HGNC Symbol;Acc:HGNC:6150] | 7067 | -0.085 | -0.2818 | No |
| 33 | RTF1 | "RTF1 homolog, Paf1/RNA polymerase II complex component [Source:HGNC Symbol;Acc:HGNC:28996]" | 7597 | -0.127 | -0.3181 | No |
| 34 | BMP4 | bone morphogenetic protein 4 [Source:HGNC Symbol;Acc:HGNC:1071] | 7816 | -0.142 | -0.3294 | No |
| 35 | HDAC1 | histone deacetylase 1 [Source:HGNC Symbol;Acc:HGNC:4852] | 7911 | -0.148 | -0.3309 | No |
| 36 | LDB1 | LIM domain binding 1 [Source:HGNC Symbol;Acc:HGNC:6532] | 8522 | -0.195 | -0.3707 | No |
| 37 | NANOG | Nanog homeobox [Source:HGNC Symbol;Acc:HGNC:20857] | 8579 | -0.200 | -0.3672 | No |
| 38 | MMP9 | matrix metallopeptidase 9 [Source:HGNC Symbol;Acc:HGNC:7176] | 8698 | -0.209 | -0.3682 | No |
| 39 | SMAD4 | SMAD family member 4 [Source:HGNC Symbol;Acc:HGNC:6770] | 9035 | -0.235 | -0.3851 | No |
| 40 | HNF1B | HNF1 homeobox B [Source:HGNC Symbol;Acc:HGNC:11630] | 9072 | -0.237 | -0.3786 | No |
| 41 | EPB41L5 | erythrocyte membrane protein band 4.1 like 5 [Source:HGNC Symbol;Acc:HGNC:19819] | 9269 | -0.253 | -0.3838 | No |
| 42 | ITGA7 | integrin subunit alpha 7 [Source:HGNC Symbol;Acc:HGNC:6143] | 9724 | -0.294 | -0.4076 | No |
| 43 | PELO | pelota mRNA surveillance and ribosome rescue factor [Source:HGNC Symbol;Acc:HGNC:8829] | 9806 | -0.302 | -0.4020 | No |
| 44 | HOXC11 | homeobox C11 [Source:HGNC Symbol;Acc:HGNC:5123] | 9907 | -0.312 | -0.3975 | No |
| 45 | ARC | activity regulated cytoskeleton associated protein [Source:HGNC Symbol;Acc:HGNC:648] | 10364 | -0.357 | -0.4190 | No |
| 46 | LAMC1 | laminin subunit gamma 1 [Source:HGNC Symbol;Acc:HGNC:6492] | 10642 | -0.387 | -0.4253 | Yes |
| 47 | SOX17 | SRY-box transcription factor 17 [Source:HGNC Symbol;Acc:HGNC:18122] | 10843 | -0.410 | -0.4247 | Yes |
| 48 | POU5F1 | POU class 5 homeobox 1 [Source:HGNC Symbol;Acc:HGNC:9221] | 10898 | -0.416 | -0.4125 | Yes |
| 49 | MMP15 | matrix metallopeptidase 15 [Source:HGNC Symbol;Acc:HGNC:7161] | 11003 | -0.430 | -0.4036 | Yes |
| 50 | LAMB1 | laminin subunit beta 1 [Source:HGNC Symbol;Acc:HGNC:6486] | 11135 | -0.448 | -0.3962 | Yes |
| 51 | COL6A1 | collagen type VI alpha 1 chain [Source:HGNC Symbol;Acc:HGNC:2211] | 11204 | -0.457 | -0.3835 | Yes |
| 52 | INHBA | inhibin subunit beta A [Source:HGNC Symbol;Acc:HGNC:6066] | 11237 | -0.462 | -0.3677 | Yes |
| 53 | DUSP2 | dual specificity phosphatase 2 [Source:HGNC Symbol;Acc:HGNC:3068] | 11768 | -0.544 | -0.3876 | Yes |
| 54 | BPTF | bromodomain PHD finger transcription factor [Source:HGNC Symbol;Acc:HGNC:3581] | 11870 | -0.565 | -0.3732 | Yes |
| 55 | COL7A1 | collagen type VII alpha 1 chain [Source:HGNC Symbol;Acc:HGNC:2214] | 11925 | -0.577 | -0.3546 | Yes |
| 56 | COL4A2 | collagen type IV alpha 2 chain [Source:HGNC Symbol;Acc:HGNC:2203] | 11940 | -0.580 | -0.3328 | Yes |
| 57 | PAX9 | paired box 9 [Source:HGNC Symbol;Acc:HGNC:8623] | 12028 | -0.600 | -0.3160 | Yes |
| 58 | DUSP4 | dual specificity phosphatase 4 [Source:HGNC Symbol;Acc:HGNC:3070] | 12068 | -0.612 | -0.2949 | Yes |
| 59 | MMP14 | matrix metallopeptidase 14 [Source:HGNC Symbol;Acc:HGNC:7160] | 12286 | -0.672 | -0.2853 | Yes |
| 60 | LAMA3 | laminin subunit alpha 3 [Source:HGNC Symbol;Acc:HGNC:6483] | 12449 | -0.724 | -0.2694 | Yes |
| 61 | ITGA5 | integrin subunit alpha 5 [Source:HGNC Symbol;Acc:HGNC:6141] | 12474 | -0.737 | -0.2422 | Yes |
| 62 | SOX2 | SRY-box transcription factor 2 [Source:HGNC Symbol;Acc:HGNC:11195] | 12485 | -0.742 | -0.2137 | Yes |
| 63 | NOTCH1 | notch receptor 1 [Source:HGNC Symbol;Acc:HGNC:7881] | 12555 | -0.776 | -0.1885 | Yes |
| 64 | DUSP5 | dual specificity phosphatase 5 [Source:HGNC Symbol;Acc:HGNC:3071] | 12721 | -0.882 | -0.1666 | Yes |
| 65 | FN1 | fibronectin 1 [Source:HGNC Symbol;Acc:HGNC:3778] | 12820 | -1.005 | -0.1346 | Yes |
| 66 | CTNNB1 | catenin beta 1 [Source:HGNC Symbol;Acc:HGNC:2514] | 12866 | -1.119 | -0.0939 | Yes |
| 67 | DUSP1 | dual specificity phosphatase 1 [Source:HGNC Symbol;Acc:HGNC:3064] | 12884 | -1.202 | -0.0479 | Yes |
| 68 | ZFP36L1 | ZFP36 ring finger protein like 1 [Source:HGNC Symbol;Acc:HGNC:1107] | 12888 | -1.244 | 0.0009 | Yes |
Table: GSEA details [plain text format]

  

Fig 2: GOBP\_ENDODERM\_DEVELOPMENT      
 Blue-Pink O' Gram in the Space of the Analyzed GeneSet

  

Fig 3: GOBP\_ENDODERM\_DEVELOPMENT: Random ES distribution      
 Gene set null distribution of ES for **GOBP\_ENDODERM\_DEVELOPMENT**
